# Long non-coding RNA LINC00997 silencing inhibits the progression and metastasis of colorectal cancer by sponging miR-512-3p

**DOI:** 10.1080/21655979.2021.1882164

**Published:** 2021-02-11

**Authors:** Zhiliang Shi, Chenglong Shen, Cheng Yu, Xiaoling Yang, Jiazhe Shao, Jian Guo, Xinguo Zhu, Guoqiang Zhou

**Affiliations:** a Department of Gastrointestinal Surgery, Changshu No. 2 Hospital, Suzhou, Jiangsu, China; b Department of General Surgery, The First Affiliated Hospital of Soochow University, Suzhou, Jiangsu, China

**Keywords:** Colorectal cancer, metastasis, migration, linc00997, miR-512-3p

## Abstract

We aimed to study the role of LINC00997 in the metastasis of colorectal cancer (CRC). LINC00997 and miR-512-3p expression in the primary colorectal cancer (NCRC) tissues and metastatic colorectal cancer (MCRC) tissues were detected using RT-qPCR. The Cancer Genome Atlas database was used to evaluate LINC00997 levels in the NCRC and MCRC tissues, and the correlations of LINC00997 expression with distant metastasis (M), regional lymph node metastasis (N), age and tumor stage were analyzed. Subsequently, RT-qPCR was performed to determine the expression of metastasis-related genes in MCRC tissues and analyze the correlation of LINC00997 or miR-512-3p level with the protein expression of metastasis-related genes. *In vitro*, LINC00997 expression in several CRC cell lines was examined. After LINC00997 silencing, cell invasion and migration were evaluated with Transwell and wound healing assays, respectively. The expression of metastasis- and EMT-related proteins was measured. Additionally, the potential interaction between LINC00997 and miR-512-3p was verified using a luciferase reporter assay. Rescue assays were conducted to clarify the regulatory effects of LINC00997 and miR-512-3p on CRC development. Results revealed that LINC00997 was frequently overexpressed in MCRC tissues, which was positively related to the tumor metastasis and stage. Additionally, LINC00997 was significantly elevated in CRC cells and LINC00997 silencing inhibited the invasion, migration and EMT of CRC cells, which was restored by miR-512-3p inhibitor. Luciferase reporter assay confirmed that LINC00997 could target miR-512-3p. In conclusion, LINC00997 regulated the metastasis of CRC by targeting miR-512-3p, providing some insights into the regulatory mechanism of CRC.

## Graphical Abstract

**Figure:**
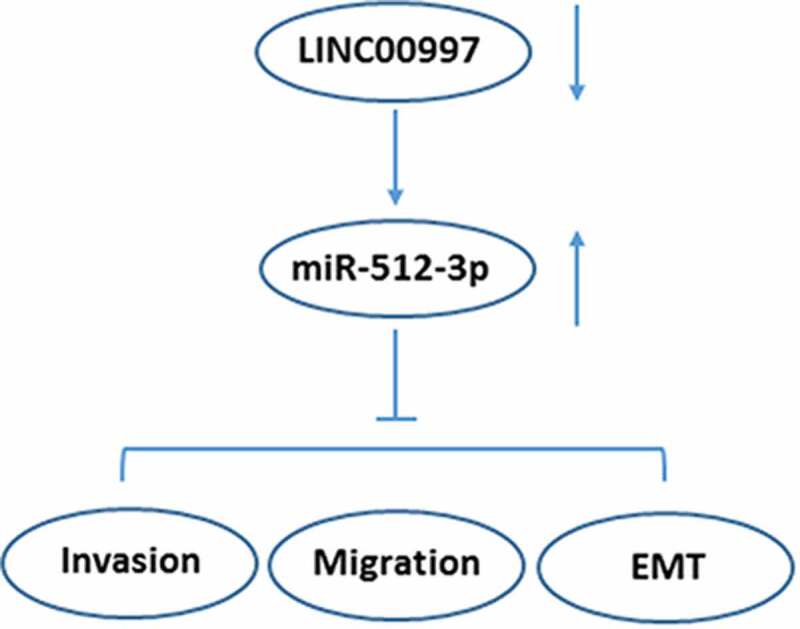

## Introduction

Colorectal cancer (CRC), is a malignant tumor in digestive system and an important health challenge for global cancer-related mortality [1,2]. It has been reported that 1–2 million CRC new cases are diagnosed annually and that each year, more than 0.6 million people die from this disease around the world [3]. Although much advancement on therapeutic strategies for CRC has been obtained in the past decade, the prognosis of CRC patients remains dismal [4]. Recurrence and metastasis of CRC are common causes for poor prognosis and high fatality rate in patients [5]. Therefore, it is desirable to explore the molecular mechanisms underlying the progression of CRC in order to identify promising prognostic biomarkers and developing effective strategies in the clinical treatment of CRC.

Long non-coding RNAs (lncRNAs), a newly discovered type of noncoding RNAs that are more than 200 nucleotides in length, have no capability to encode the functional proteins [6]. Accumulating studies have confirmed that lncRNAs exert a diverse set of functions in multiple biological processes, such as growth, differentiation and metastasis via interaction with microRNAs (miRNAs/miRs) or proteins. A large number of lncRNAs have been reported to be abnormally expressed in the progression of many cancers, indicating that they may serve as potential mediators in carcinogenesis [7–9]. LINC00997, a new found long noncoding RNA, whose expression in kidney renal clear cell carcinoma metastases was higher than in nonmetastatic tumors, promotes the metastasis in kidney renal clear cell carcinoma [10]. However, the expression and function of LINC00997 in CRC remain elusive so for. This study unveils the key regulatory crosstalk between LINC00997 and miRNAs in cancer.

In this study, the expression of LINC00997 in nonmetastatic colorectal cancer (NCRC) tissues, metastatic colorectal cancer (MCRC) tissues and several CRC cell lines was detected. Then, the effects of LINC00997 on the metastasis of CRC and the potential molecular mechanisms were explored. These results might provide a promising prognostic biomarker and therapeutic target for the treatment of CRC.

## Materials and methods

### Clinical sample collection

NCRC tissues, matched adjacent normal tissues, MCRC tissues and the corresponding adjacent non-tumor tissues (N = 8 in each group) were obtained from CRC patients who received surgical treatment at the Department of Gastrointestinal Surgery, Changshu No. 2 Hospital. All patients who had not received chemotherapy or radiotherapy prior to the operation were enrolled in the study. These experiments were approved by the Ethics Committee of the Changshu No. 2 Hospital (Approval number 2019-KY-015), and written informed consent was obtained from all patients. All samples collected from surgery were immediately snap frozen in liquid nitrogen and stored at −80°C.

### Cell culture

Several human CRC cell lines (SW480, SW1417, SW620, Caco2, LS174T, HCT15, LoVo, HT29 and HCT116) and the normal intestinal epithelial cell line (HIEC) used in this study were obtained from American Type Culture Collection (Manassas, VA, USA). These cells were cultured in DMEN (Gibco BRL, Grand Island, NY, USA) containing 10% FBS (HyClone Laboratories, Logan, UT, USA). The incubator was humidified and maintained at a temperature of 37°C, with 5% CO_2_ and 95% room air.

### Cell transfection

HCT116 cells in logarithmic phase were collected and loaded into 6-well plates (1x10^6^ per well), which were then incubated at 37°C until 85% confluence. A total of two short hairpin (sh)RNAs targeting LINC00997 (shRNA-LINC00997-1 or shRNA- LINC00997-2), a scrambled shRNA [shRNA-negative control (NC)], miR-512-3p inhibitor, miR-512-3p inhibitor NC (inhibitor-NC), miR-512-3p mimic and scrambled miRNA control (mimic-NC) were designed and chemically synthesized by Shanghai GenePharma Co., Ltd. (Shanghai, China). Cell transfections were conducted with Lipofectamine 2000® reagent (Invitrogen; Thermo Fisher Scientific, Inc.) following the product instructions. At 48 h after post-transfection, cells were collected and the transfection efficiency was evaluated by means of RNA extraction and reverse transcription-quantitative polymerase chain reaction (RT-qPCR).

### Transwell assay

Transwell assay was employed to determine the ability of cell invasion. Briefly, after transfection, 3 × 10^4^ HCT116 cells resuspended in 100 μl serum-free DMEM medium were placed in the upper chamber coated with Matrigel (BD, USA). The lower chamber was filled with DMEM containing 10% FBS as a chemoattractant. After 24 h incubation, the non-invading cells that remained in the upper chamber were removed. Cells that migrated through Matrigel were fixed with methanol and stained with crystal violet. The number of invading cells in five randomly selected visual fields were counted under an inverted microscope (Olympus Corporation, Tokyo, Japan; 100x) and the mean was calculated.

### Scratch wound healing assay

For wound healing assay, cells (6x10^5^ cells per well) were plated into six-well plates and incubated at 37°C to achieve 80% confluence. Then, serum-free medium was utilized to incubate overnight prior to initiating the experiment. The monolayer was scratched with a sterilized 100 μl pipette tip. After 48 h, cell migration was photographed using an inverted microscope (Olympus; magnification, 100x) and the distance was determined and normalized to the 0 h control as the relative migration rate for comparison.

### Immunofluorescence assay

HCT116 cells were plated on the coverslips in 24-well plates and cultured until 80% confluence was reached. Subsequently, cells were fixed in 4% paraformaldehyde at room temperature for 20 min, followed by the addition of 0.05% Triton X-100 solution at room temperature for 10 min. The cells were blocked with 5% normal goat serum, cells were incubated with primary antibodies (Cell Signaling Technology; Boston, MA, USA). Then cells were incubated with DyLight™ 488-conjugated secondary antibody (Thermo Fisher Scientific, Inc.) and stained with 4ʹ, 6-diamidino-2-phenylindole (Sigma-Aldrich; Merck KGaA). After mounting, the cells were observed under a fluorescence microscope (Olympus Corporation).

### Luciferase reporter assay

Potential target genes of LINC00997 were predicted via an online bioinformatics software (http://starbase.sysu.edu.cn/), and the potential interaction between miR-512-3p and LINC00997 was detected by means of luciferase reporter assay. Luciferase reporter vectors encoding the wild-type (WT) or mutant (MUT) LINC00997 were first designed. To conduct the luciferase reporter assay, cells were co-transfected with LINC00997-WT or LINC00997-MUT and miR-512-3p or mimic-NC. 48 h after transfection, luciferase activities were analyzed using the Dual-Luciferase Reporter Assay System (Promega, Madison, WI). The ratios of luminescence from Firefly to Renilla luciferase were normalized through three independent experiments.

### RT-qPCR

Total RNA was extracted from patient specimens and CRC cell lines with TRIzol reagent (Invitrogen; Thermo Fisher Scientific, Inc.) in accordance with the specification provided by the supplier. Total RNA was reverse transcribed by means of a Reverse Transcription kit (Beijing TransGen Biotech, Beijing, China). MiRNA expression was detected in triplicate using SYBR PrimeScriptTM miRNA RT-PCR Kit (Takara Biotech). qPCR was then performed with 2 μg cDNA, as the template using iTaq™ Universal SYBR® Green Supermix (Bio-Rad Laboratories, Inc.) on an ABI 7500 instrument (Applied Biosystems; Thermo Fisher Scientific, Inc.). All values were normalized to GAPDH or U6 which was used as the internal reference gene. 2^−ΔΔCt^ method was applied for calculation of relative expression levels.

### Western blot analysis

Cells were lysed in RIPA (Donghuan Biotech, Shanghai, China). A BCA protein detection Kit (Donghuan Biotech) was used to measure the protein concentrations in accordance with the manufacturer’s protocol. The equivalent amount of protein extract was separated on 10% SDS-PAGE, followed by transferred onto PVDF membranes (Millipore, Madison, WI, USA). Then, the blots were blocked in PBS containing 5% nonfat milk for 2 h, followed by incubation with primary antibodies (Cell Signaling Technology; Boston, MA, USA). After the membranes were incubated with the immunoreactive protein bands were visualized using the Odyssey Infrared Imaging System (LI-COR Biosciences). Protein bands were analyzed using ImageJ software (National Institutes of Health). GAPDH was used as an internal control.

### Statistical analysis

All data were presented as means ± standard deviation (SD). Comparisons between two groups were made by Student’s t-test with GraphPad Prism 6.0 software. One-way ANOVA with Tukey’s post-hoc test was employed to perform the multi-sample analysis. The correlations were analyzed by Spearman’s rank test. P-values of <0.05 were taken as statistically significant.

## Results

### LINC00997 is highly expressed in MCRC tissues and is associated with clinicopathological features

**Figure 1.**
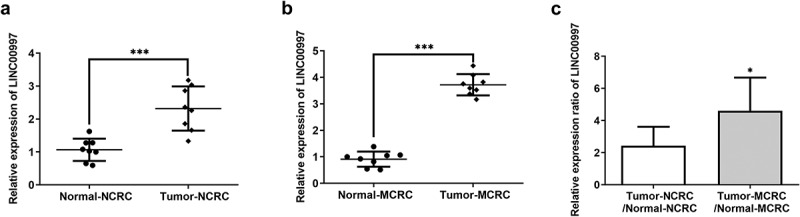
LINC00997 is highly expressed in MCRC tissues. (a) The expression of LINC00997 in NCRC tissues and the corresponding adjacent tissues was determined using RT-qPCR assay. ***P < 0.001. (b) LINC00997 level in MCRC tissues and para-carcinoma tissues was detected with RT-qPCR. ***P < 0.001. (c) The relative expression ratio of LINC00997 in the Tumor-MCRC/Normal-MCRC group and Tumor-CRC/Normal-NCRC group. *P < 0.05 vs. Tumor-NCRC/Normal-NCRC

**Figure 2.**
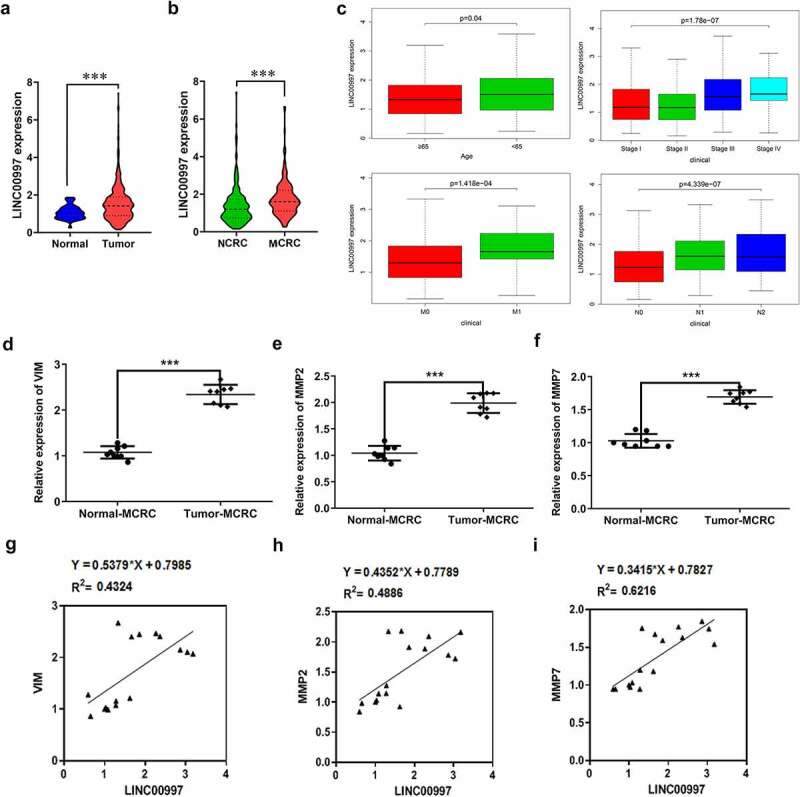
LINC00997 expression is associated with the clinicopathological features of CRC. (a) The Cancer Genome Atlas database (TCGA) was used to evaluate the expression of LINC00997 in CRC tumor tissues and adjacent non-tumor tissues. (b) LINC00997 expression in the MCRC and NCRC tissues was tested using the TCGA database. (c) The correlation of LINC00997 expression with distant metastasis (m), regional lymph node metastasis (n), age and tumor stage was analyzed using TCGA database. (d-f) RT-qPCR was employed to assess the expression of VIM, MMP2 and MMP7 in the MCRC tissues and the corresponding beside tissues. ***P < 0.001. (g-i) The correlation between LINC00997 expression and VIM, MMP2 and MMP7 level

LncRNAs play significant roles in the progression of CRC. Emerging evidence supports LINC00997 expression in kidney renal clear cell carcinoma metastases was higher than that in nonmetastatic tumors, which promotes the metastasis in kidney renal clear cell carcinoma [10]. In this study, firstly, the expression of LINC00997 in NCRC and MCRC tissues as well as the corresponding adjacent tissues was determined by RT-qPCR assay. As shown in Figure 1a, LINC00997 level was notably elevated in the NCRC tissues relative to the para-carcinoma tissues. Additionally, notably upregulated LINC00997 level was observed in the MCRC tissues compared with the corresponding adjacent tissues (Figure 1b). As presented in Figure 1c, the relative expression ratio of LINC00997 in the Tumor-MCRC/Normal-MCRC group was higher than that in the Tumor-CRC/Normal-NCRC group. Moreover, results from The Cancer Genome Atlas (TCGA) database exhibited in Figure 2a indicated that LINC00997 level was markedly enhanced in the tumor tissues compared with the normal tissues. Importantly, there is significant difference between tissues in the MCRC group and NCRC group (Figure 2b). Furthermore, it was obviously found that LINC00997 level was positively correlated with the age, stage, distant metastasis (M) and regional lymph node metastasis (N) (Figure 2c). Subsequently, the expression of genes related to metastasis including VIM, matrix metalloproteinase 2 (MMP2), and MMP7 was examined with RT-qPCR. As presented in Figure 2d–f, markedly upregulated expression of VIM, MMP2 and MMP7 was noticed in the MCRC tissues as compared to the adjacent tissues. As observed in Figure 2g–i, there are obvious positive correlations between LINC00997 expression and VIM, MMP2 and MMP7 levels, respectively. These observations reveal that LINC00997 is remarkably upregulated in MCRC tissues and is associated with the clinicopathological features.

### LINC00997 silencing inhibits migration, invasion and epithelial-mesenchymal transition (EMT) of CRC cells

**Figure 3.**
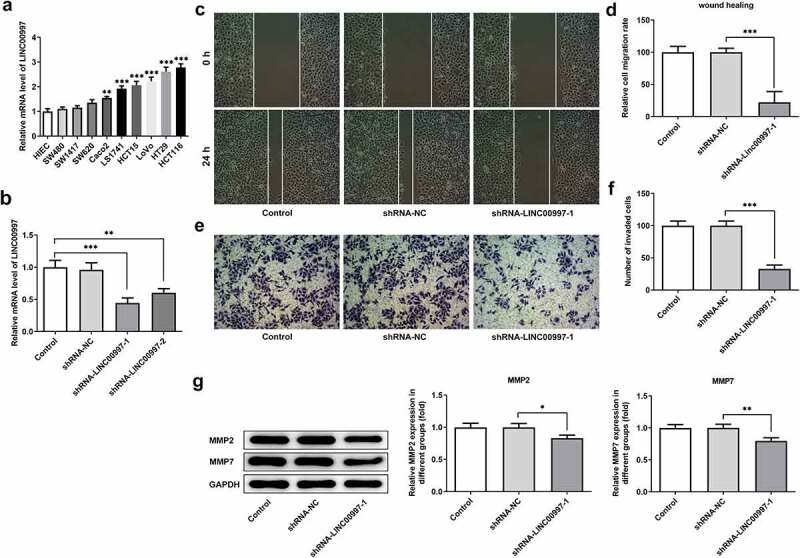
LINC00997 silencing suppressed the migration and invasion of CRC cells. (a) LINC00997 level in several CRC cell lines was detected using RT-qPCR. **P < 0.01, ***P < 0.001 vs. HIEC. (b) The level of LINC00997 was tested with RT-qPCR after transfection with shRNA-LINC00997 into HCT116 cells. **P < 0.01, ***P < 0.001. (c and d) Cell migration was tested using wound healing assay. (e and f) The invasive ability of HCT116 cells was determined with Transwell assay. (g) Western blot analysis was used to examine the expression of MMP2 and MMP7. *P < 0.05, **P < 0.01, ***P < 0.001

**Figure 4.**
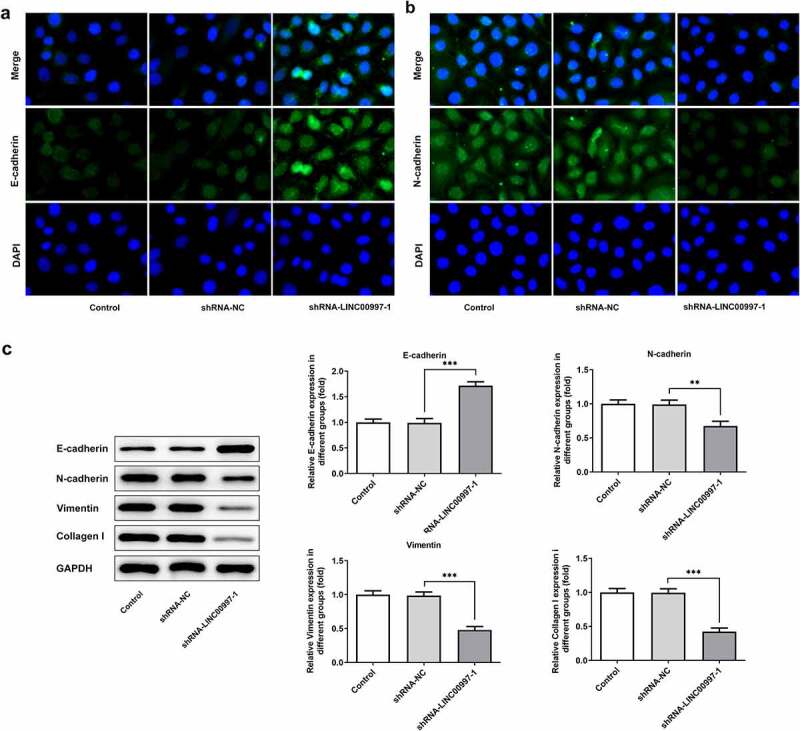
LINC00997 silencing inhibited the epithelial-mesenchymal transition (EMT) of HCT116 cells. The expression of (a) E-cadherin and (b) N-cadherin in HCT116 cells was evaluated using immunofluorescence assay. (c) The expression of EMT-related proteins was measured by means of western blot analysis. **P < 0.01, ***P < 0.001

To further explore the role of LINC00997 in the metastasis of CRC, its expression in several CRC cell lines was examined using RT-qPCR. As exhibited in Figure 3a, LINC00997 level was notably increased in the CRC cell lines relative to the HIEC cells, especially in the HCT116 cells, and this cell line was used to perform the following experiments. Then, LINC00997 was silenced by transfection with shRNA-LINC00997, and significantly reduced LINC00997 expression is found in Figure 3b. As shown in Figure 3c–f, LINC00997 silencing dramatically restrained the migration and invasion of HCT116 cells as compared to those cells in the empty vector group. Moreover, the expression levels of metastasis-related proteins MMP2 and MMP7 displayed the same changing tendency with the results of migration and invasion of HCT116 cells (Figure 3g). Concurrently, it was noticed that E-cadherin expression was conspicuously upregulated in the HCT116 cells, accompanied by downregulated expression of N-cadherin, Vimentin and Collagen I (Figure 4a–c). Therefore, it could be concluded that LINC00997-downregulation restrains the migration, invasion and EMT of CRC cells.

### MiR-512-3p can be directly targeted by LINC00997

**Figure 5.**
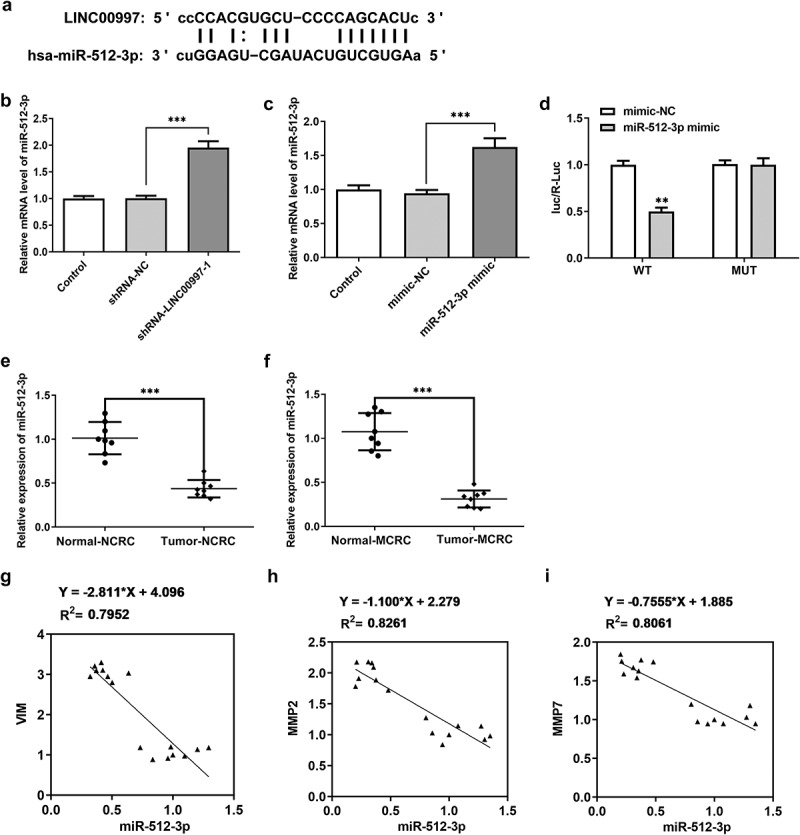
MiR-512-3p could be directly targeted by LINC00997. (a) Binding region between LINC00997 and miR-512-3p. (b) RT-qPCR was employed to evaluate the expression of miR-512-3p after LINC00997 silencing. (c) The expression of miR-512-3p was assessed after transfection with miR-512-3p mimic. (d) A luciferase reporter assay was performed to determine the relative luciferase activity. **P < 0.01 vs. mimic-NC. (e) The expression of miR-512-3p in NCRC tissues and the corresponding adjacent tissues was determined using RT-qPCR assay. ***P < 0.001. (f) MiR-512-3p level in MCRC tissues and para-carcinoma tissues was detected with RT-qPCR. ***P < 0.001. (g-i) The correlations between LINC00997 expression and VIM, MMP2 and MMP7 levels

To further explore the regulatory mechanisms of LINC00997 in CRC, potential target genes of LINC00997 were predicted using an online bioinformatics software. The results from the Starbase analysis revealed that miR-512-3p was one of the potential targets of LINC00997 (Figure 5a). And the level of miR-512-3p was notably upregulated after LINC00997 silencing (Figure 5b). As shown in Figure 5c, miR-512-3p was notably elevated after transfection with miR-512-3p mimic. The dual-luciferase reporter assay demonstrated the binding of miR-512-3p with LINC00997, since the miR-512-3p mimic + LINC00997 WT group exhibited lower luciferase activity as compared to the mimic-NC group (Figure 5d). Subsequently, miR-512-3p expression in NCRC and MCRC tissues was tested by RT-qPCR. As exhibited in Figure 5e and f, miR-512-3p level was dramatically decreased in both NCRC and MCRC tissues relative to the para-carcinoma tissues. As is observable in Figure 5g–i, there are significant negative correlations between miR-512-3p expression and VIM, MMP2 and MMP7 level, respectively. These observations reveal that miR-512-3p could be directly targeted by LINC00997.

### MiR-512-3p-knockdown restores the impact of LINC00997 silencing on the migration, invasion and EMT in HCT116 cells

**Figure 6.**
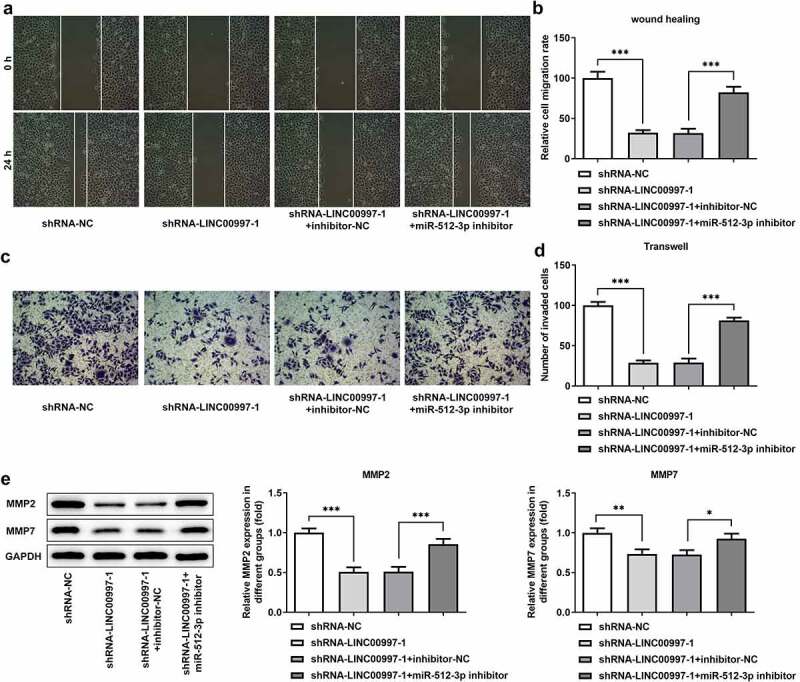
MiR-512-3p knockdown restored the inhibitory effects of LINC00997 silencing on the migration and invasion of HCT116 cells. (a and b) Wound healing assay was utilized for the evaluation of cell migration. (c and d) Transwell assay was applied for the measurement of cell invasion. (e) The expression of MMP2 and MMP7 was examined using western blotting. *P < 0.05, **P < 0.01, ***P < 0.001

**Figure 7.**
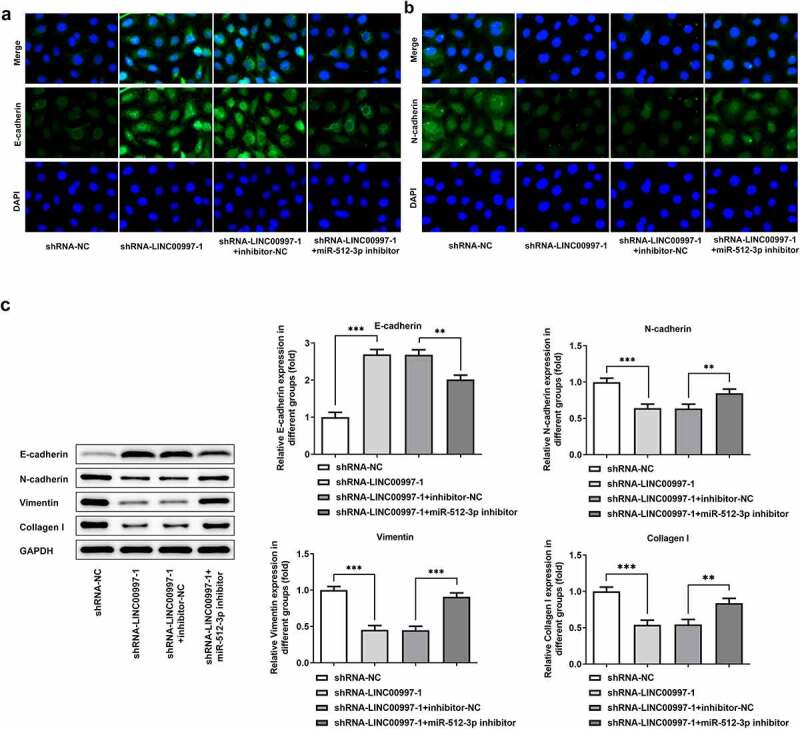
MiR-512-3p knockdown attenuated the impacts of LINC00997-downregulation on the EMT of HCT116 cells. The level of (a) E-cadherin and (b) N-cadherin in HCT116 cells was detected with immunofluorescence assay. (c) The expression of EMT-related proteins was measured using western blot analysis. **P < 0.01, ***P < 0.001

To investigate the regulatory association between LINC00997 and miR-512-3p in the metastasis of CRC, cell migration and invasion were examined when HCT116 cells were co-transfected with shRNA-LINC00997 and miR-512-3p inhibitor. As exhibited in Figure 6a–d, co-transfection of shRNA-LINC00997 and miR-512-3p inhibitor restored the impacts of LINC00997 silencing alone on the migration and invasion of HCT116 cells. Consistently, the levels of MMP2 and MMP7 presented the same changing trend with the migration and invasion (Figure 6e). Furthermore, the addition of miR-512-3p inhibitor alleviated the inhibitory effects of LINC00997-downregulation on the levels of EMT-related proteins including E-cadherin, N-cadherin, Vimentin and Collagen I (Figure 7a–c). These data provide evidence that LINC00997 regulates the migration, invasion and EMT of HCT116 cells by sponging miR-512-3p.

## Discussion

CRC is currently one of the prevalent cancer types worldwide. Under most circumstances, symptoms of cancer are detected at an advanced stage, leading to poor prognosis [11]. Although considerable progress has been made in the treatment of CRC recently, the prognosis of patients with CRC remains dismal [12]. Therefore, finding new therapeutic strategies for CRC is of great significance. Continuing advances in the development of next-generation sequencing and transcriptomics suggest that the investigation of lncRNAs in the regulation of cancer has emerged as a potential research field [13]. Worldwide research has focused on identifying potential diagnostic and prognostic markers, which might predict survival outcomes accurately. A growing body of reports demonstrate that aberrant expression of lncRNAs was involved in the development and progression of different types of cancer, including CRC [14,15]. Findings in the current study firstly demonstrated that LINC00997 was dramatically increased in CRC tissues and cell lines, and LINC00997-knockdown might repress the metastasis of CRC via inhibiting migration, invasion and EMT of CRC cells partly by sponging miR-512-3p.

Metastasis is the hallmark of the malignant biological behavior of CRC, and interdiction of these progresses is a crucial factor to improve biomedical treatment worldwide [16]. As migration and invasion of cancer cells play key roles in tumor metastasis [17], we explored whether these two features were involved in LINC00997-mediated tumor-promoting action. Previous reports have indicated that lncRNAs participate in every stage of carcinogenesis and tumor progression, such as tumor growth, tumor metastasis and tumor angiogenesis [18,19]. Emerging evidence supports that LINC00997 expression in kidney renal clear cell carcinoma metastases was higher than that in nonmetastatic tumors, which promotes the metastasis in kidney renal clear cell carcinoma [10]. In the current study, significantly elevated LINC00997 expression was observed in both NCRC and MCRC tissues, and there is significant difference between Tumor-MCRC/Normal-MCRC and Tumor-NCRC/Normal-NCRC groups. Importantly, LINC00997 level was positively correlated with the age, stage, distant metastasis (M), regional lymph node metastasis (N) and the expression of metastasis-associated proteins, suggesting the conspicuous role of LINC00997 in the progression MCRC. Of note, LINC00997 silencing suppressed the migration and invasion of CRC cells.

Increasing research has validated that EMT, a crucial driver of tumor progression, is the classical tumor metastasis theory [20,21]. Cells undergoing EMT lose the typical epithelial phenotype and are converted into a mesenchymal phenotype with enhanced abilities of migration and invasion, which results in cancer metastasis [22]. Aberrant expression of a large number of genes was found during the activation of EMT. E-cadherin, a crucial epithelial marker, was notably downregulated, whereas there were increases in the expression of N-cadherin and Vimentin, which are crucial mesenchymal marker genes [23,24]. A growing body of reports reveal that lncRNAs could modulate the invasion and metastasis of CRC cells through the regulation of the EMT process. For instance, lncRNA ADAMTS9-AS1, as a prognostic marker, promotes cell proliferation and EMT in CRC [25]. LncRNA-differentiation antagonizing non-protein coding RNA (lncRNA-DANCR) contributes to the metastasis of CRC through activation of EMT process [26]. The present study revealed that LINC00997 knockdown suppressed the EMT progress.

Previous study has highlighted the importance of lncRNAs as competing endogenous RNAs in tumor biology [27]. lncRNAs could modulate gene expression by sponging specific miRNAs, a type of noncoding RNA molecules of 19–25 nucleotides, via indirectly mediating gene expression at the post-transcriptional level [28]. The starbase database predicted that miR-512-3p can be targeted by LINC00997, which was verified using a luciferase activity reporter assay in this study. Study has proven that miR-512-3p overexpression promotes the inhibition of metastasis in non-small cell lung cancer [29]. MiR-512-3p was reported to inhibit breast cancer cell growth and metastasis [30]. Above-mentioned research prompted further investigation into the role of LINC00997 and miR-512-3p in the metastasis of CRC. The present study revealed that miR-512-3p was dramatically reduced in MCRC tissues, which was negatively correlated with genes related to metastasis. The effect of LINC00997 silencing on migration, invasion and EMT was restored by a miR-512-3p inhibitor. These data provide evidence that LINC00997 regulates the metastasis of CRC by sponging miR-512-3p.

Overall, LINC00997 silencing suppresses migration, invasion and EMT of CRC cells via regulation of miR-512-3, which presents a new promising target for the clinical diagnosis and therapeutic interventions of CRC.

## Conclusion

Taken together, this study for the first time found that LINC00997 was overexpressed in MCRC tissues and cells. Mechanistically, LINC00997 knockdown suppresses the migration, invasion and EMT of CRC cells by sponging miR-512-3p. Our findings provide a new regulatory mechanism consisting of LINC00997/miR-512-3p in CRC for researchers in this field, identifying a new theoretical basis for targeted therapy.

## Data Availability

All data generated or analyzed during this study are included in this published article.

## Highlights

(1) LINC00997 is frequently overexpressed in MCRC tissues.

(2) LINC00997-upregulation is linked to clinicopathological features.

(2) LINC00997 silencing inhibits migration, invasion and EMT of CRC cells.

(3) LINC00997 acts as a sponge of miR-512-3p.
